# Contributing factors for reduction in maternal mortality ratio in India

**DOI:** 10.1038/s41598-024-65009-0

**Published:** 2024-06-27

**Authors:** Himanshu Tolani, Sutapa Bandyopadhyay Neogi, Anuj Kumar Pandey, Pijush Kanti Khan, Sidharth Sekhar Mishra

**Affiliations:** International Institute of Health Management Research, New Delhi, India

**Keywords:** Maternal mortality ratio, SRS, NFHS, Heatmap, Bayesian, Spatio-temporal, Sustainable development goals, Health care, Medical research, Risk factors, Mathematics and computing

## Abstract

Maternal mortality ratio (MMR) estimates have been studied over time for understanding its variation across the country. However, it is never sufficient without accounting for presence of variability across in terms of space, time, maternal and system level factors. The study endeavours to estimate and quantify the effect of exposures encompassing all maternal health indicators and system level indicators along with space–time effects influencing MMR in India. Using the most recent level of possible -factors of MMR, maternal health indicators from the National Family Health Survey (NFHS: 2019–21) and system level indicators from government reports a heatmap compared the relative performance of all 19 SRS states. Facet plots with a regression line was utilised for studying patterns of MMR for different states in one frame. Using Bayesian Spatio-temporal random effects, evidence for different MMR patterns and quantification of spatial risks among individual states was produced using estimates of MMR from SRS reports (2014–2020). India has witnessed a decline in MMR, and for the majority of the states, this drop is linear. Few states exhibit cyclical trend such as increasing trends for Haryana and West Bengal which was evident from the two analytical models i.e., facet plots and Bayesian spatio- temporal model. Period of major transition in MMR levels which was common to all states is identified as 2009–2013. Bihar and Assam have estimated posterior probabilities for spatial risk that are relatively greater than other SRS states and are classified as hot spots. More than the individual level factors, health system factors account for a greater reduction in MMR. For more robust findings district level reliable estimates are required. As evident from our study the two most strong health system influencers for reducing MMR in India are Institutional delivery and Skilled birth attendance.

## Introduction

A maternal death is “the death of a woman while pregnant or within 42 days of termination of pregnancy, irrespective of the duration and site of pregnancy, from any cause related to or aggravated by the pregnancy or its management but not from accidental or incidental causes”^1^. The likelihood of a woman dying while pregnant is quantified by maternal mortality ratio (MMR), which is the number of maternal deaths per 100,000 live births within a given time period. Globally during 2017 about 295,000 women died during and following pregnancy and childbirth^2^. Most of these deaths occurred in low and middle-income countries (LMIC), and most could have been prevented.

Maternal mortality reduction has always been a global health priority. Sustainable Development Goals (SDG) target reduction of global MMR to less than 70 per 100,000 births, with no country having MMR more than twice the global average^3^. Achieving this SDG target would necessitate an average reduction of roughly three times the yearly rate reduction achieved during the (Millennium Development Goals) MDG era^4^. Despite a documented progress towards this SDG target^5,6^, a greater acceleration is warranted.

The "three delay" concept^7^ i.e., delays in making the decision to seek care, delays in getting to a health facility, and delays in health institutions providing proper care, has been the guiding framework behind formulating all policies and programs to reduce MMR. These critical time periods are predictive of most obstetric complications^7^. Most of the indicators for tracking such delays fall under the ambit of various demographic sample surveys; however, the inadequacy of data collection at smallest spatial unit makes it more difficult to gather routine and comprehensive information about the maternal death.

The Sample Registration system (SRS), a demographic sample survey in India uses nationally representative sample to offer reliable estimates of maternal mortality for major states^8^. National Family and Health Survey (NFHS) is another large-scale, multi-round survey conducted in a representative sample of households throughout states and districts of India. The survey offers information on fertility, infant and child mortality, family planning, maternal and child health, reproductive health, nutrition, anaemia, and the use and quality of health and family planning services for India at the national, state and district levels^9^.

India is a confederation of thirty-six (36) states/ union territories (UTs) having diverse and heterogenous culture, geographies, health infrastructure, and health indicators. It acknowledges the regional variations and inequalities and is cognizant of its commitment to cater to the needs of the entire people. Understanding variation for any event or a health outcome through one or two dimensions is never sufficient without accounting for presence of variability across in terms of space and time. We generally look upon the socio-economic exposures for explaining the outcome under study but seldom account for variation in spatial and temporal dimensions. Decline in maternal mortality is associated with ample of variates: socio-economic variables, increased government health expenditure, employment of government schemes, progress in institutional delivery, antenatal and postnatal care, nutrition, birth preparedness and contraception use are some of the indicators which have improved significantly over the years.

Emphasising on the reliability of data for major states from SRS we aim to evaluate evidence for spatio-temporal variations accounting for 45% decline in MMR since last decade across nineteen (19) SRS states. Thus, the study proposes to identify the time of the transition for significant decline in maternal mortality across 19 SRS states between 2000 and 2020 and to understand linear/cyclical declining pattern. The study also endeavours to estimate the spatial risk of individual states and later categorise states as hot spots based on posterior probabilities obtained through adopted model for selected states in India.

## Methods

The study used secondary data available in public domain viz SRS and NFHS. The sampling design adopted for SRS is a unit-stage stratified simple random sample without replacement except in stratum II (larger villages) of rural areas, where two stage stratification has been applied^10^. Total sample units covered by SRS (2018–2020) is 8841 and total population covered is 83,10,000^11^.

NFHS-5 used a multistage stratified sampling, as part of which the census enumeration blocks (CEBs) in urban areas and villages in rural areas were selected as the primary sampling units (PSUs) based on Probability Proportional to Size (PPS) sampling. It covers 636,699 households, 724,115 women (15–49 years) and 101,839 men (15–54 years)^9^.

To explore the trends of MMR, data were taken from SRS published reports for 19 states for five time periods 2014–16^12^, 2015–17^13^, 2016–18^14^, 2017–19^11^ and 2018–20^11^. Spatial categorisation of 19 SRS states is visualised in supplementary Fig. 1. In order to explore the spatial risk of different states, we retrieved maternal health related indicators from the most recent NFHS-5 (2019–21) survey.

The analysis includes health system level or macro level indicators such as Health index, Government Health Expenditure (GHE), Out of Pocket Expenditure (OOPE), Janani Suraksha Yojna (JSY) beneficiary, Number of Accredited Hospitals, Number of National Quality Assurance Standards (NQAS) certified hospitals. The case definitions of these factors are described in Supplementary table 1. Apart from the above indicators, various health systems centric maternal health indicators were also considered such as percentage of women receiving antenatal care (ANC), percentage of women receiving postnatal care (PNC) within 48 h of delivery, percentage of women with birth order greater than 3, percentage of women with birth interval less than two years, percentage of children who were delivered with skilled birth attendance (either from doctor or auxiliary nurse mid-wife or from any health professional), percentage of women using any contraception method, percentage of women with body mass index (BMI) less than 18.5 kg/m^2^, percentage of women with anaemia, proportion of women who delivered through caesarean section were considered for analysis^15^.

Geo-statistical models focus on the response-determinant dynamics to visualize the extent of uncertainty or the degree of regularity for the underlying physical process. These hidden uncertainties might be associated with the data, the underlying spatio-temporal process or with the model parameters which are accounted for by assigning probability distributions to them. A section under the model will explain utility of spatial and spatio-temporal effects in explaining MMR across 19 SRS states of India.

Individual trend of MMR for states was analysed through simple linear regression visualisation using ggplot2^16^ package in R which provides a useful collection of tools to partition the data using "facets"^17^, which separate the data frame based on the values of specific columns. We obtained graphs each with a separate linear regression line with 95% Confidence Interval for coefficients of indicators fitted against time.

By summarizing massive data sets, the data visualisation approach known as the heatmap enhances descriptive analysis and enhances visualisation, specifically when there is a wealth of data available for many time periods, locations, or qualities. Each data point received after standardisation is given a relative score, and each score is then further assigned to a colour based on our preference. Heatmap scales down each feature under study by standardisation over the span where span can be in years or set of places depending on the data. Scaling in heatmap is done by standardizing the observations in a particular column. The mathematical formula for standardizing is same as standardizing a normal variable to standard normal variable.

The notations ${\text{O}}_{\text{it}}$ and ${\text{E}}_{\text{it}}$ denote observed and expected maternal mortality ratio in area i = 1,2,3…. N (N is the number of states as in SRS report; N = 19 at time t = 1,…,T. In this study T = 5 (SRS-2014–2016, SRS-2015–17, SRS-2016–18, SRS-2017–19 and SRS-2018–20).

$${\text{O}}_{\text{it}} \sim \text{ Poisson}({\text{E}}_{\text{it}}{\uprho }_{\text{it}})$$

where ${\uprho }_{\text{it}}$ represent risk of maternal mortality in district i at time t. ${\text{E}}_{\text{it}}$ can be computed as follows.

${\text{E}}_{{{\text{it}}}} = \mathop \sum \limits_{{{\text{t}} = 1}}^{{\text{T}}} {\text{P}}_{{{\text{it}}}} \times {\text{~}}\frac{{\mathop \sum \nolimits_{{{\text{i}} = 1}}^{{\text{N}}} {\text{O}}_{{{\text{it}}}} }}{{\mathop \sum \nolimits_{{{\text{i}} = 1}}^{{\text{N}}} {\text{P}}_{{{\text{it}}}} }}$ where ${\text{P}}_{\text{it}}$ is the population under study for state i at time t.

Since MMR is number of maternal deaths per lakh live births, it is standardised with respect to population in each state; therefore, we took E as 1 for each state.

We propose the model^18^ for explaining Maternal Mortality risk variation across 19 SRS states

1 $${\text{log}}({\rho }_{it}) =\alpha +{{\text{s}}_{i}}+{{\text{u}}_{i}}+\upgamma {{\text{time}}_{t}}+{\updelta }_{i}{{\text{time}}_{t}}$$

#SPTM.

Both spatially structured random effect (${\text{s}}_{\text{i}}$) and spatio-temporal effect (${\updelta }_{\text{i}}$) are assigned as Conditionally Autoregressive (CAR) Distribution^18^ as a prior distribution. Model (1) is the baseline model for our study and is described as a model with purely spatial and spatio-temporal random effects. In model 1, estimated log-relative risk is given by $\text{log}{(\uprho }_{\text{it}})$ at base e and is explained by an overall global risk (given by $\alpha$); a risk related to the spatial location (${\text{s}}_{\text{i}}$) that can be attributed to factors associated to particular region; a temporal risk trend common to all areas $(\upgamma )$ that can be attributed to changes in coding the underlying factors, policies affecting the whole country and finally an indicator of adjusted area specific trend $({\updelta }_{\text{i}}$) that reflect particular effects of each state. More specifically, ${\updelta }_{\text{i}}$ is difference between area specific trend and overall trend $\upgamma$. Adjusted area specific trends are usually of more interest in planning government intervention strategies as their probability of being greater or smaller than some threshold indicates hot-spots and cold spots respectively. The Poisson distribution is the fundamental distribution when discussing discrete events. Spatial random effects when captured through CAR distribution handles both overdispersion and spatial autocorrelation when counts are modelled through Poisson distribution^18^.

In order to adjust all the 17 exposures discussed as indicators along with the space–time effects we constructed and analysed two different models separated based on health system level factors and individual maternal health factors.

2 $$\begin{aligned}{\text{log}}({\rho }_{1\text{it}}) & = {\alpha }_{2}+ {\upbeta }_{1}*{{\text{Anemia}}_{\text{i}}}+{\upbeta }_{2}*{\text{Low BMI}}+{\upbeta }_{3}*{{\text{Low B. Int}}_{\text{i}}}+{\upbeta }_{4}*{{\text{Contraception}}_{\text{i}}}\\ &\quad+{\upbeta }_{5}*{{\text{C. Section}}_{\text{i}}}+{{\upbeta }_{6}*{{\text{Birth Order}}_{\text{i}}}+{\text{s}}_{1\text{i }}}+{{\text{u}}_{1\text{i }}}+{\upgamma }_{1}{{\text{time}}_{\text{t}}}+{{\updelta }_{1\text{i}}}{{\text{time}}_{\text{t}}}\end{aligned}$$

#Maternal Health factors + SPTM.

3 $$\begin{aligned}{\text{log}}({\uprho }_{2\text{it}}) & = {\alpha }_{3}+ {\upbeta }_{7}*{{\text{ANC}}_{\text{i}}}+{\upbeta }_{8}*{{\text{PNC}}_{\text{i}}}+{\upbeta }_{9}*{{\text{Institutional}}_{\text{i}}}+{\upbeta }_{10}*{{\text{Health Index}}_{\text{i}}}\\ & \quad +{{\upbeta }_{11}*{{\text{Accrediction}}_{\text{i}}}+{\upbeta }_{12}*{{\text{NQAS}}_{\text{i}}}+{\upbeta }_{13}*{{\text{GHE}}_{\text{i}}}+{\upbeta }_{14}*{{\text{OOPE}}_{\text{i}}}+{\upbeta }_{15}*{{\text{JSY}}_{\text{i}}}} \\ & \quad {+{\upbeta }_{16}*{{\text{Population}}_{\text{i}}}+{\upbeta }_{17}*{{\text{Skilled Attendance}}_{\text{i}}}+{\text{s}}_{2\text{i }}}+{{\text{u}}_{2\text{i }}}+{\upgamma }_{2}{{\text{time}}_{\text{t}}}+{\updelta }_{2\text{i}}{{\text{time}}_{\text{t}}}\end{aligned}$$

#Health system level Indicators + SPTM.

Model 2 explains MMR risk through maternal health indicators along with spatial and spatio-temporal effects. Model 3 explains MMR risk through health system level indicators along with spatial and spatio-temporal effects.

Relative risks computed as spatial risk for each areal unit is calculated as ${\widehat{\uprho }}_{\text{i}}=\text{exp}\left({\widehat{\text{s}}}_{\text{i}}\right)$ associated to region $\text{i}.$ Posterior probability that the spatial risk for MMR is greater than 1, i.e., ${\text{p}}={\text{P}}({\widehat{\uprho }}_{\text{i}}>1/ {\text{Y}}_{\text{it}})$.

All the parameters based on model 1, 2 and 3 are estimated and analysed through Integrated Nested Laplace Approximation (INLA) and the goodness and complexity of different models is assessed and compared through Deviance Information Criterion^19^ (DIC).

### Integrated nested Laplace approximation

An alternate method for approximating Bayesian estimates apart from Monte Carlo Markov Chain (MCMC) technique that is faster and more computationally efficient was introduced^20^ in 2009 with the name of Integrated Nested Laplace Approximation (INLA). Computing high-dimensional integrals is the main step in Bayesian inference and in our adpated model we estimate our posterior marginals through R-INLA. Analysis for estimates of MMR and covariates have handled in R version 4·1·3. Bayesian estimates have been computed through R-INLA. Facet plots and Heatmaps are obtained in R version 4·1·3.

## Results

**Figure 1:**
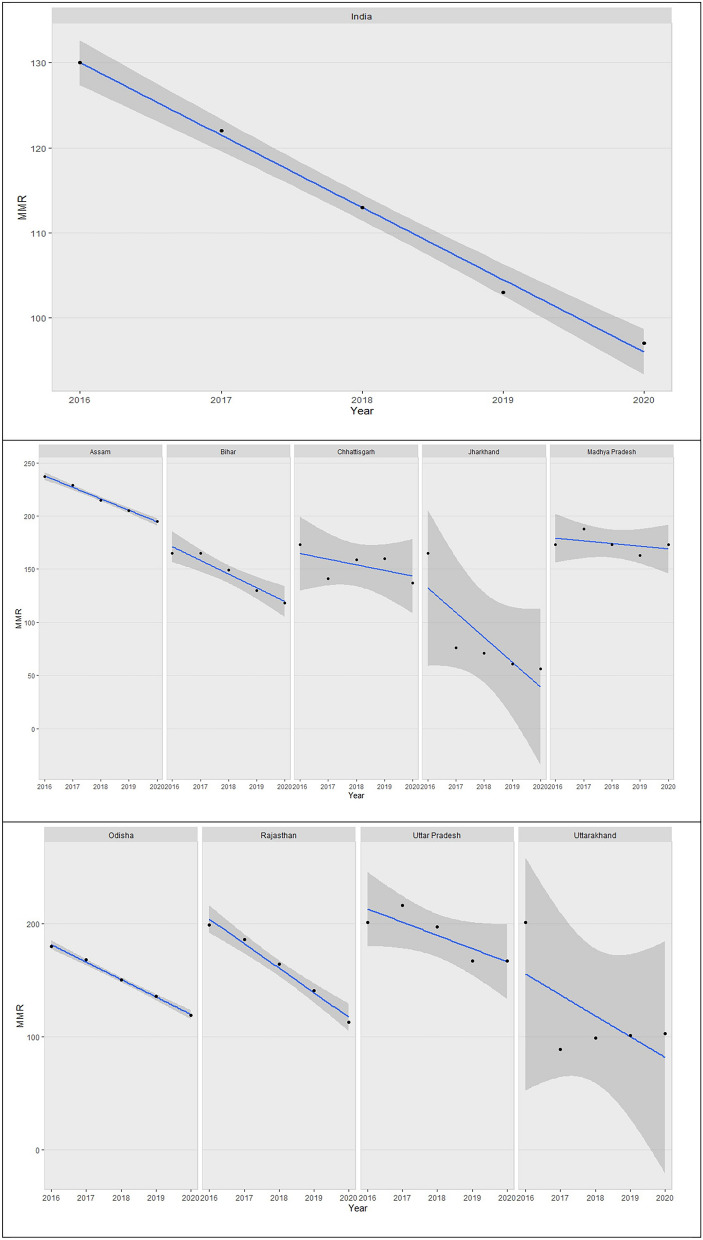

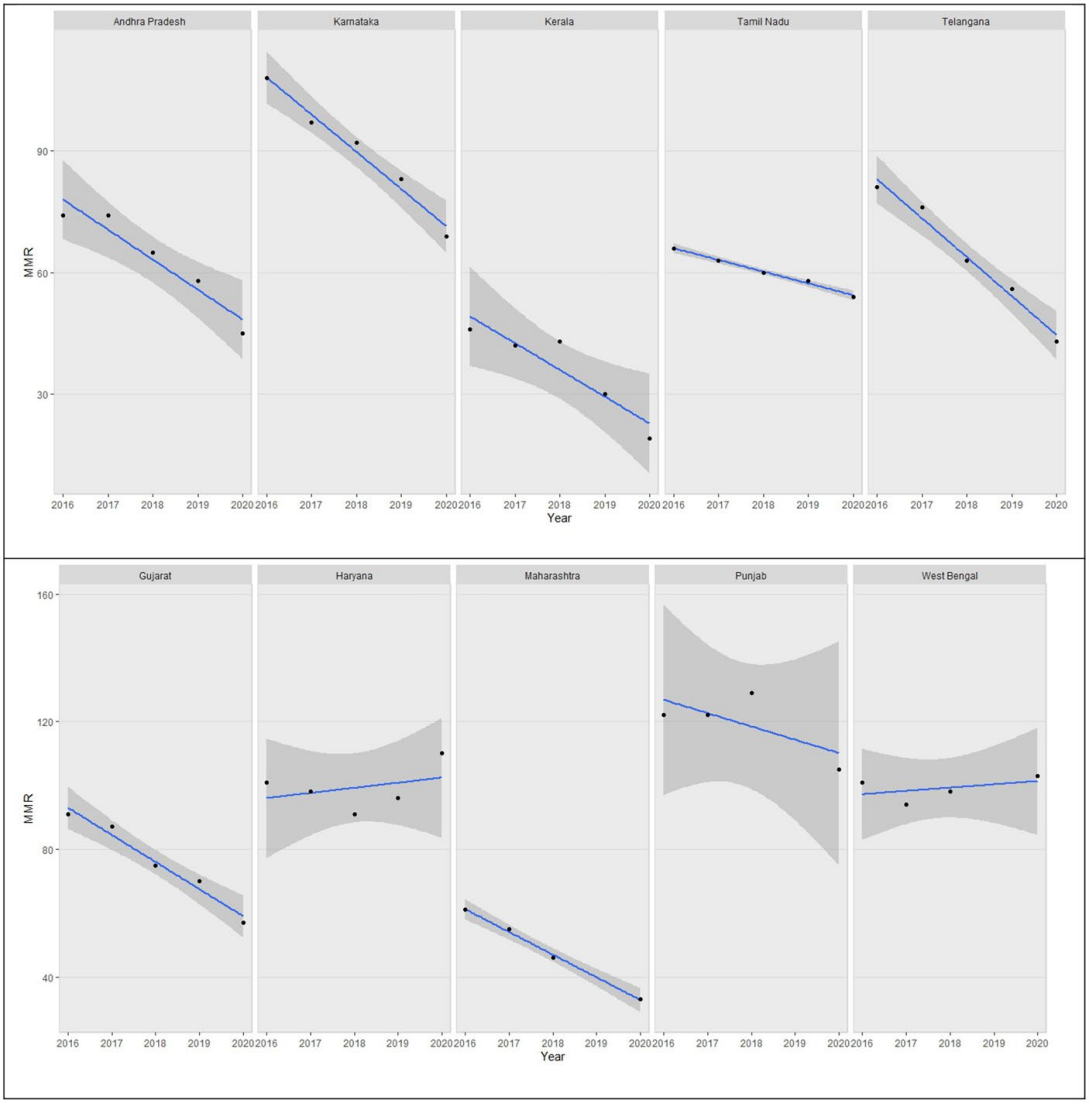
Trends for MMR in India and SRS States.

Facet plots for trend of MMR from 2016 to 2020 across 19 SRS states in India (Fig. 1) shows an overall declining trend for all states except for Haryana and West Bengal. States of northern part of the country like Punjab, Uttarakhand, Jharkhand, Haryana, Chhattisgarh, Madhya Pradesh, and Uttar Pradesh exhibit larger variation in MMR as indicated be grey scales representing 95% C.I. in comparison to other states. Southern states like Andhra Pradesh, Tamil Nadu, Kerala and Karnataka are amongst better performing States in context with the MMR as compared to other SRS states.

**Figure 2:**
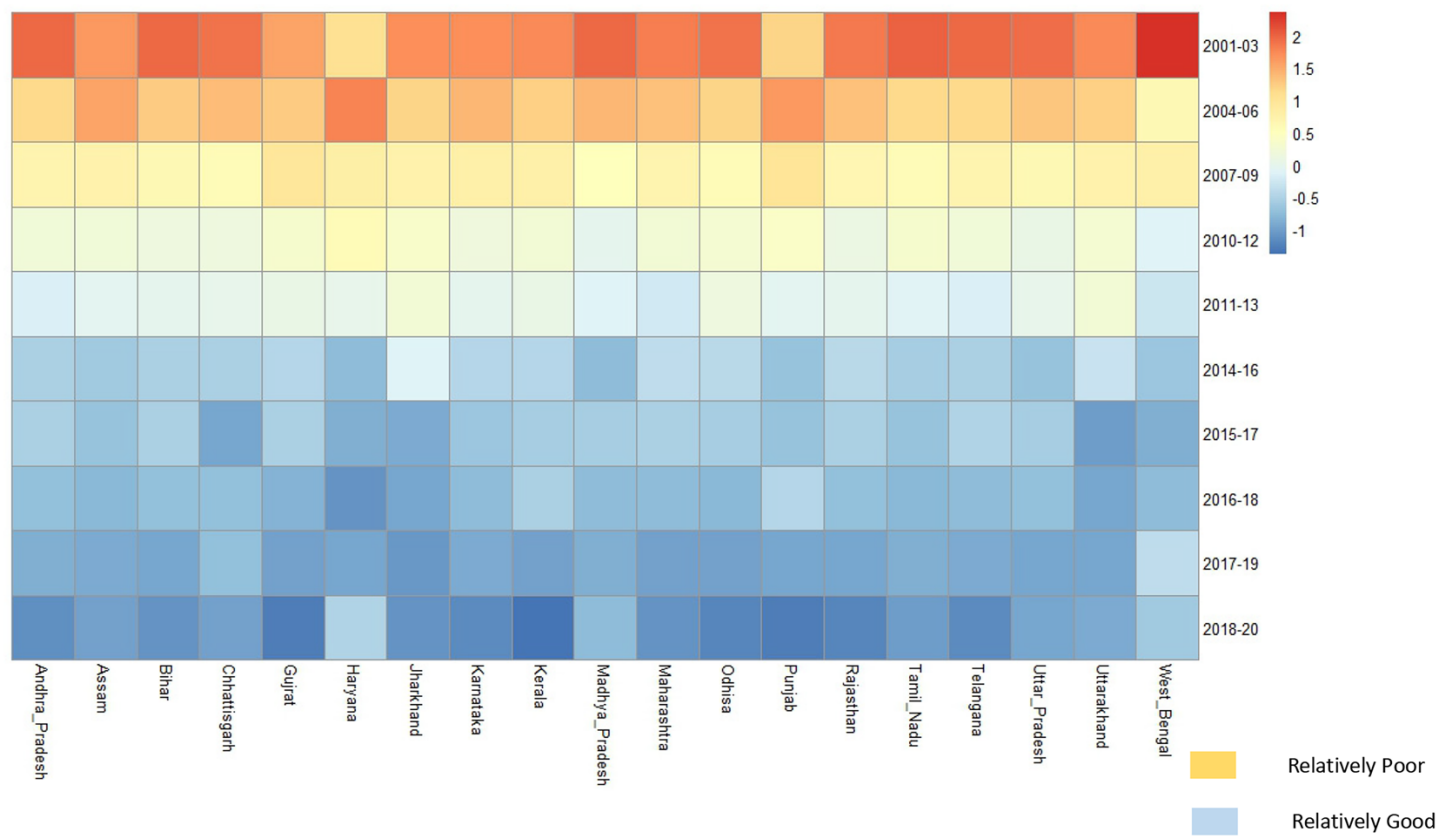
Heatmap representaing MMR trends from 2000 to 2020 in for 19 SRS states.

Heatmap in Fig. 2 represent the scaled estimates of MMR from 2001 to 2020 for 19 SRS states. This reiterates that there is considerable decline in MMR over the years. It can be inferred that the period of major transition for decline in MMR was from 2010 to 2013 and from 2015–2017 is common for all states. During 2016–2020, few states have shown a slight increase in MMR relative scale such as Haryana and West Bengal.

**Figure 3:**
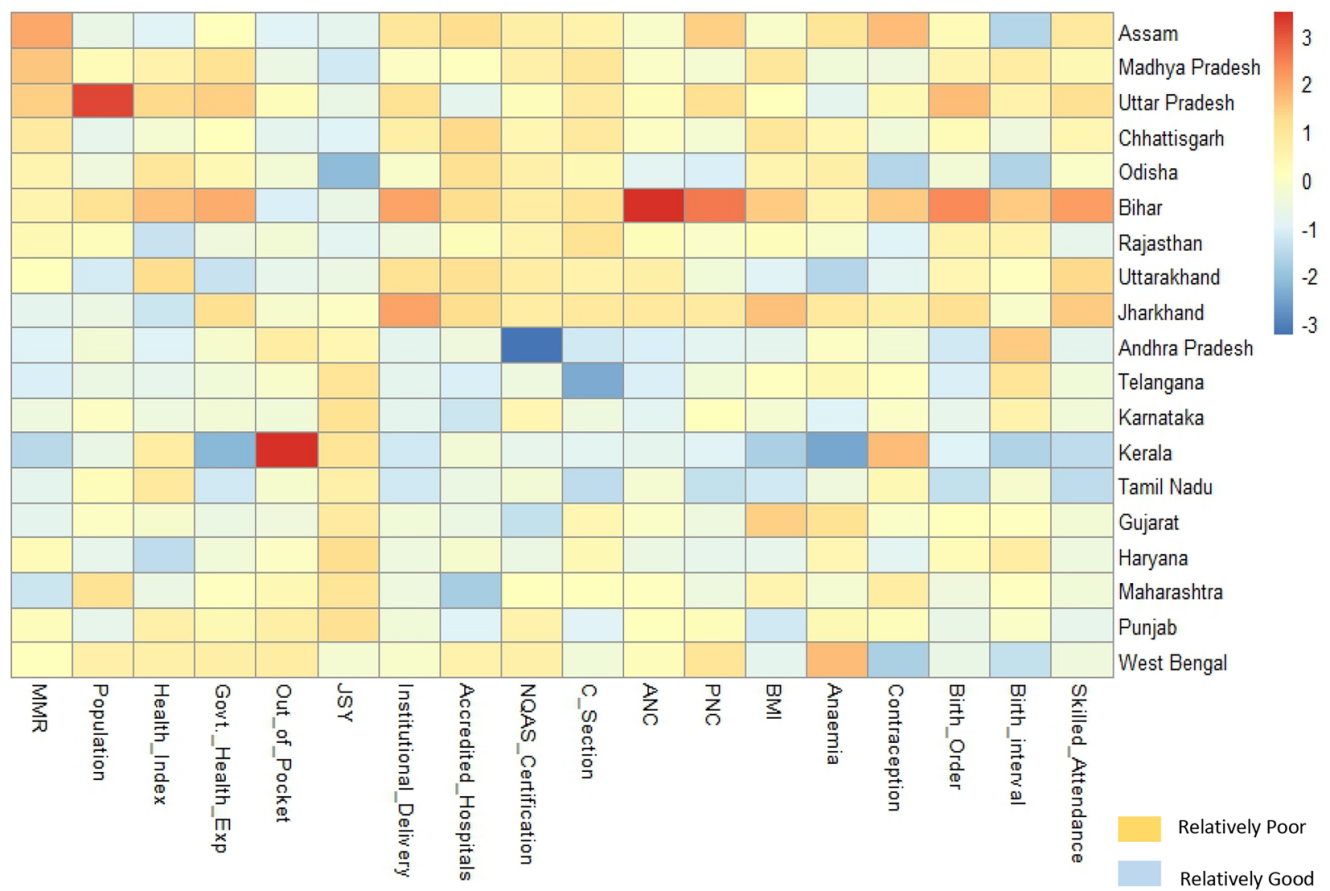
Heatmap for MMR and associated covariates for the States of India, 2019–21.

Figure 3 presents the scaled level MMR and associated maternal and health systems level covariates in India. It gives us an essence of performance of the associated covariates with MMR among 19 SRS states. The government health expenditure is relatively lower for states like Madhya Pradesh, Uttar Pradesh, Bihar, Chhattisgarh, and Punjab. Out of pocket expenditure is relatively higher for states like Kerala, West Bengal, Punjab, Maharashtra, Andhra Pradesh and Uttar Pradesh. There seems to be a poor corelation between the percentage of women receiving any money through JSY and proportion of institutional deliveries. States where both JSY beneficiaries and institutional deliveries are low includes Jharkhand and West Bengal. Southern states have fared better in terms of accreditation and NQAS certification of health facilities.

Utilization of ANC services are relatively better in most of the states except Bihar, Jharkhand and Uttarakhand. PNC services remains poorer in many states like Bihar, Assam, Jharkhand, Uttar Pradesh and West Bengal. These states also lag behind in skilled attendance at birth.

Table 1 presents posterior summaries of all parameters investigated under three scenarios i.e., model 1, 2 and 3. We found evidence for significant decline in terms of overall mean time trend from 2014 to 2020 whose posterior mean was estimated as − 0·121 and is significant as per 95% credible interval (C.I.) Estimates for model adequacy and model complexity i.e., DIC of model 3 being lowest is claiming to be best fit among all the three models. Model 2 gives the posterior estimates of maternal health indicators and the intensity of their influence on MMR. Positive posterior estimates in Table 1 indicate increase in MMR and negative posterior estimates indicate decrease in MMR. For example, proportion of women having birth interval less than two years or proportion of low birth interval in any SRS state have higher likelihood of MMR with relative risk of $\text{exp}(\widehat{{\upbeta }_{3})}=1.638695$. Similarly, proportion of women with birth order greater than 3 or higher birth order has quite similar effect on MMR i.e., $\text{exp}\left(\widehat{{\upbeta }_{6}}\right)=$ 1.499003. The two health system influencers of MMR which are responsible to decrease MMR strongly are institutional delivery and Skilled birth attendance with relative risk $\text{exp}(\widehat{{\upbeta }_{9})}=0.4867523$ and $\text{exp}(\widehat{{\upbeta }_{17})}=0.379842$ respectively. Increase in health system indicators like NQAS with exp $\left(\widehat{{\upbeta }_{12}}\right)=$ 0.82037, accreditation status with exp ($\widehat{{\upbeta }_{11})}=$ 0.959829, Health Index with $\text{exp}(\widehat{{\upbeta }_{10})}=$ 0.615082 and GHE with $\text{exp}(\widehat{{\upbeta }_{13}})=$ 0.920351 indicate decrease in MMR. OOPE has relative risk $\text{exp}(\widehat{{\upbeta }_{14})}=$ 1.29706 and JSY has $\text{exp}(\widehat{{\upbeta }_{15})}=$ 1.386247. The implementation of JSY was on high focus states which is the reason for higher likelihood of MMR in states where larger proportion of women received JSY.

**Table 1:** Posterior Summary for regression parameter estimates ($\alpha ,{\beta }{\prime}\text{s and }\gamma$).

	Mean	95% C.I	Mean	95% C.I	Mean	95% C.I	RR
Intercept ($\widehat{\alpha }$)	2·345	(2·081, 2·533)	0·0020	(− 0·005, 0·009)	0.4311	(0.193, 0.640)	
Overall trend ($\widehat{\upgamma }$)	− 0·167	(− 0·234, − 0·135)	− 0·030	(− 0·038, − 0·017)	− 0.121	(− 0.142, − 0.10)	
Individual factors							
Birth Order > 3 $(\widehat{{\upbeta }_{6})}$			0·4048	(0·199, 0·6211)			1.49900267
Birth interval < 2 $(\widehat{{\upbeta }_{3})}$			0·4939	(0·476, 0·511)			1.638695
C-Section $\widehat{{(\upbeta }_{5})}$			− 0·125	(− 0·235, 0·140)			0.8824969
Contraception $\widehat{{(\upbeta }_{4})}$			− 0·116	(− 0·266, 0·392)			0.8904752
Anemia ($\widehat{{\upbeta }_{1})}$			0·0594	(− 0·066,0.0654)			1.0612
BMI < 18.5 ($\widehat{{\upbeta }_{2})}$			0·2912	(0.182, 0.4204)			1.338032
Health systems factors							
Population $\widehat{({\upbeta }_{16})}$	0·1655	(0·011, 0·375)					1.179982963
ANC $\widehat{({\upbeta }_{7})}$	− 0·050	(− 0·058, − 0·044)					0.60653066
PNC $\widehat{({\upbeta }_{8})}$	− 0·036	(− 0·044, 0·027)					0.964640293
Inst. Delivery $\widehat{({\upbeta }_{8})}$	− 0·720	(− 0·905, − 0·4781)					0.486752
Health Index $\widehat{({\upbeta }_{10})}$	− 0·486	(− 0·811, − 0·2532)					0.615082
Accreditation $\widehat{({\upbeta }_{11})}$	− 0·041	(− 0·055, 0·302)					0.959829
NQAS $\widehat{({\upbeta }_{12})}$	− 0·198	(− 0·359, − 0·0832)					0.82037
GHE $\widehat{(13)}$	− 0·083	(− 0·847, − 0·0496)					0.920351
OOPE $\widehat{({\upbeta }_{14})}$	0·2601	(0·1989, 0·3134)					1.29706
JSY $\widehat{({\upbeta }_{15})}$	0·3266	(0·2042, 0·4406)					1.386247
Skilled Att.$\widehat{({\upbeta }_{17})}$	− 0·968	(− 1·145, − 0·8026)					0.379842
Model (3) DIC = 1781 Model (2) DIC = 1819 Model (1) DIC = 1858							

**Figure 4:**
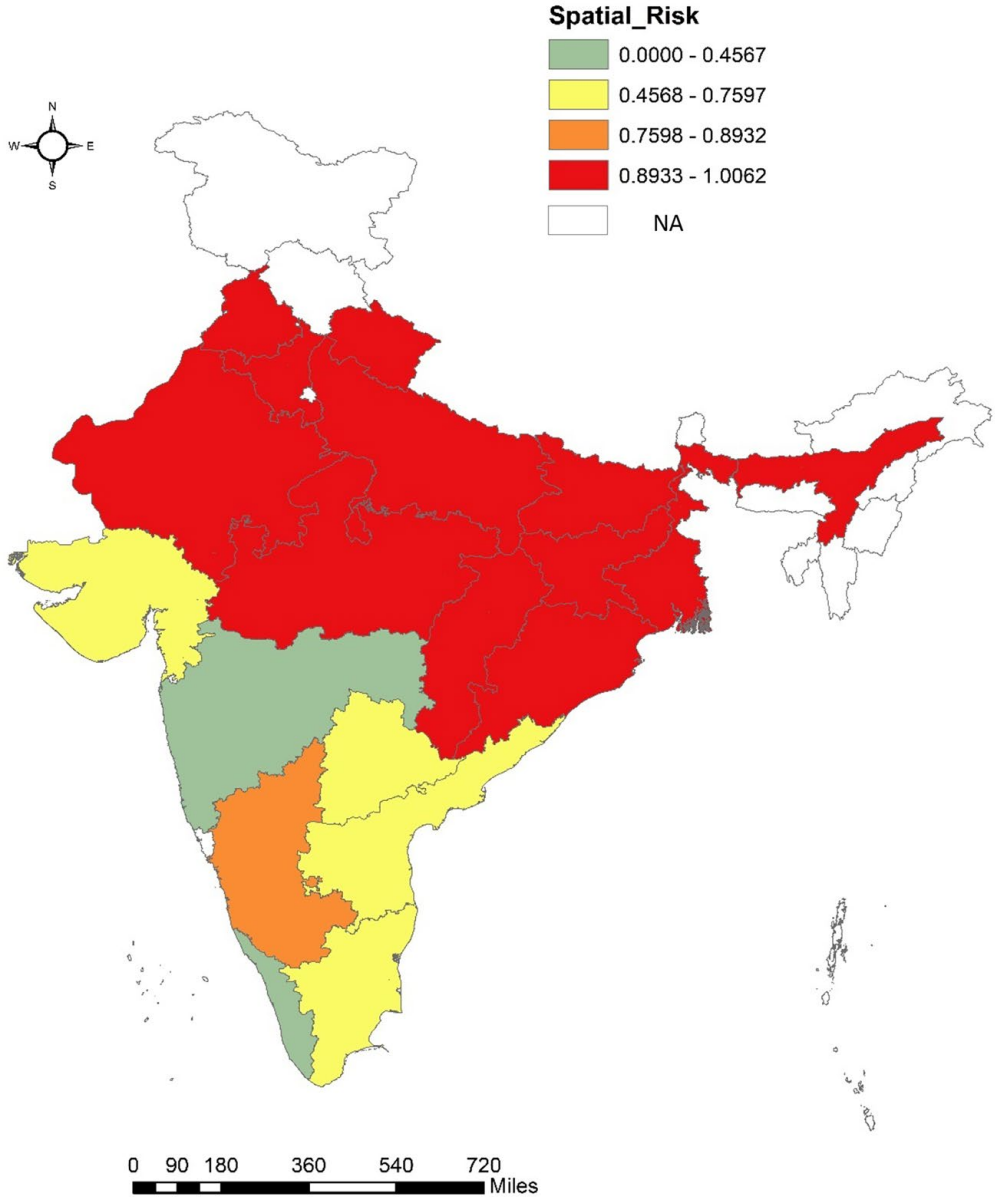
Spatial Risk of MMR for 19 SRS states.

**Figure 5:**
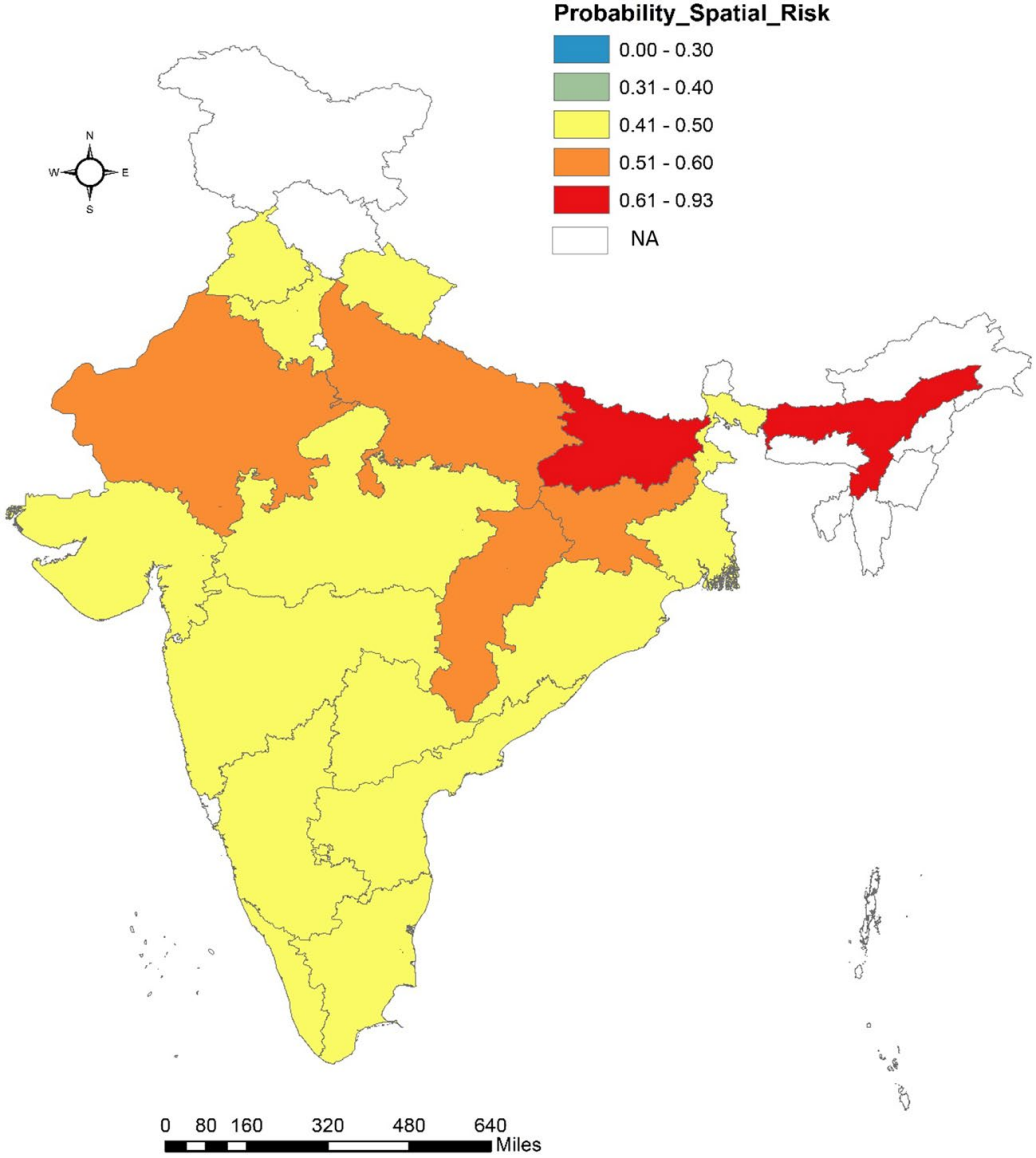
Posterior probability $$\text{P}({\widehat{\uprho }}_{\text{i}}>1/ {\text{Y}}_{\text{it}})$$.

**Figure 6:**
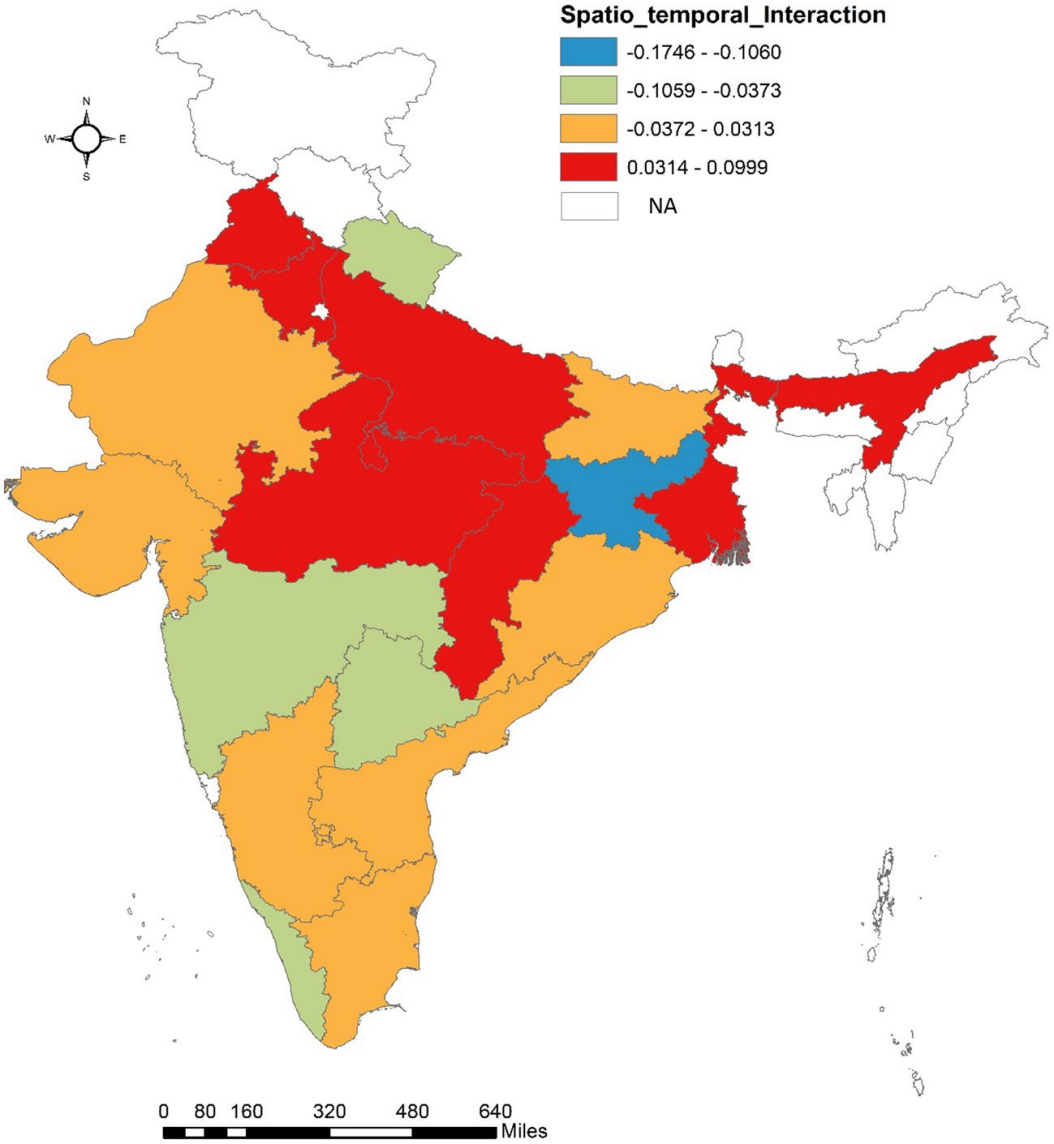
Posterior mean for spatio-temporal interaction effect ${(\updelta }_{\text{i}})$.

Spatial risk of mortality associated with each state is computed based on model 1 and is presented in Fig. 4. Spatial risk greater than 1 for a state means relative risk of MMR is high than national average level of MMR which is common to all states. Further, in order to categorise states which, have spatial risk close to 1 and might have high probability of MMR risk greater than 1, we evaluated probability of spatial risk greater than 1 for each state as depicted in Fig. 5. The spatial and spatio-temporal effects have been depicted in Figs. 4, 5 and 6 and represent the spatial variation in MMR highlighting individual impact of states in explaining MMR.

Figure 5 depicts the probability such that ${\widehat{\uprho }}_{\text{i}}>1$ is greater than or less than 0·5 i.e., if $\text{p}=\text{P}({\widehat{\uprho }}_{\text{i}}>1/ {\text{Y}}_{\text{it}})$; then we have mapped for different values of p. States for which $\text{p}>0\cdot 5$ are classified as hot-spots because they have higher likelihood of having high MMR as compared to others which have $\text{p}<0\cdot 5$. The latter are qualified as cold spots.

Figure 6 displays the posterior mean for spatio-temporal interaction effect for each SRS state. Since ${\updelta }_{\text{i}}$ is difference between area specific trend and overall mean time trend $(\upgamma )$, ${\updelta }_{\text{i}}$ provides evidence for spatial patterns because of time^21^. Positive posterior mean for ${\updelta }_{\text{i}}$ indicate risk of maternal mortality has increased over the five time periods from 2014–16 to 2018–20. Negative posterior mean indicates for declining risk of maternal mortality.

It may thus be interpreted that the states like Punjab, Haryana, Uttarakhand, UP, Rajasthan, MP, Chhattisgarh, Bihar, Jharkhand, Odisha West Bengal, and Assam have MMR more than the national average. However, the two states that should deserve focussed attention in terms of MMR reduction includes Bihar and Assam. Bihar exhibits relatively poor maternal health indicators than others which is also in line with estimate of spatial risks and computed probability. When we compare the MMR of each state relative to itself, many states including Punjab, Haryana, UP, MP, Chhattisgarh, West Bengal and Assam demonstrates higher risk over the past five years. For more robust findings district level reliable estimates are required.

## Discussion

The present study appraises spatial and spatio-temporal effects for explaining variation in MMR across 19 SRS states. India has witnessed a significant decline in MMR over the past ten years, although there are disparities between states. Most of the decline seems to be attributable to the improvement in health systems indicators across the country, as evident from Bayesian spatio-temporal analysis.

Our analysis demonstrates that the period of major transition for decline in MMR was between 2010 and 2013 and that is common for all states. This period coincides with the implementation of government initiatives such as National Health Mission in 2005 and monetary benefit schemes like JSY and JSSK to cater to the needs of pregnant women and promote institutional deliveries^22,23^. Institutional deliveries markedly increased after the implementation of JSY^22,24,25^. Reduction in maternal mortality is most sensitive to access to emergency services in equipped health facilities^26,27^.

The second transition occurred after 2015, that further coincides with the launch of various breakthrough schemes like Dakshta^28^ and Labour Room Quality Improvement Initiative (LaQshya) which is a strategic intervention to improve quality of care during and around childbirth. The National Health Mission's Pradhan Mantri Surakshit Matritva Abhiyan (PMSMA)^29^ was introduced in 2016. The programme seeks to offer guaranteed, thorough, and high-quality antenatal care to all pregnant women on the ninth of every month, free of charge. It is difficult to infer whether these transitions were strictly due to the introduction of specific large scale public health interventions, since a more granular analysis of the determinants indicate the differential uptake of services across the states.

Variation in MMR and the associated maternal level and system level influencers are computed and visualised together in one frame through heatmap. The differential decline is in line with the literature highlighting the poor performance of EAG states^30^. However, it is pertinent to note that states such as Bihar and Assam would have considerably higher MMR as compared to other states in India as evident from probabilities of spatial risk of individual states. This calls for urgent attention and better implementation of programs in these states for desired results at the national level.

Our study provides geographical and temporal perspectives of explaining variation in MMR using the Bayesian spatio-temporal approach making it one of its own kind. The study has evaluated and quantified the spatial risk, probability of spatial risk and estimate of spatio-temporal effect for each SRS state with respect to MMR for last five time periods making it more robust and generalizable.

Sufficient studies leverage spatial and spatio-temporal methods as indispensable tool for identifying inequalities in access for maternal health services and disparities in utilization^31^. Classical statistical algorithms such as Spatial autocorrelation^32^, Geographically weighted regression^33^, Local Indicators of Spatial Autocorrelation^34^ have been employed frequently for understanding maternal health and MMR integrated through panel data models in China, Ethiopia and fifty four (54) African countries respectively. Inverse distance weighted method of interpolation method has been used to assess spatial patterns of maternal mortality in Tanzania^35^. Poisson regression was utilised in understanding spatial distribution of maternal deaths in Brazil^36^. Studies done under Bayesian formulation for explaining maternal deaths and MMR are very few but have gained popularity recently due to the ease of MCMC and INLA as hassle-free convenient routes for statistical computations. Bayesian multivariable regression^32^ was employed recently to understand trends of MMR and its cause pattern in 34 Chinese provinces while a Brazilian study used Bayesian empirical model^37^ to assess covid deaths in obstetric population. The present study combines the essence of Poisson regression handled through Conditional Autoregressive distribution which counters extra-poisson variation^38^ forms the basis to proposed Bayesian spatio-temporal modelling approach.

We have attempted to explore most probable covariates for MMR. SRS and Health Management Information Systems (HMIS) are the two major data bases that provide information pertaining to maternal deaths and related indicators in India. Both these databases encompass major limitations, but we have used Bayesian methods and model parameters, which accounts for less challenges^39^, which arises when unstable estimates happen because of low counts of events in areas and high sampling variation^40–42^. This makes this study more unique in its true sense. In this study we have addressed variation in estimates of MMR at different time points. Studies in past tried to estimate district wise maternal mortality ratio through data triangulation^43^ but robust and reliable district level estimates for MMR from government of India are awaited. The presently reported HMIS data suffers from lack of quality^44^, manipulation and misreporting^45^ and hence should be refrained from use in scientific deliberations or should be used with enough caution.

Very few studies have synthesised MMR explanations across space and time in India, and it is extremely uncommon to explain MMR using the broadest range of maternal health markers. For the first time in an Indian study, evaluation of 17 maternal and health system markers that may have an impact on MMR, as well as effects of pure spatial and spatiotemporal interactions, is carried out (Fig. 4, Models 2 and 3 Table 1). Model 2 and Model 3 were designed to analyse maternal level effects and health system effects separately. Health system factors were found to explain risk of MMR better than maternal level factors (Table 1, DIC = 1781).

Despite the fact that our study has numerous methodological and conceptual strengths, it also has certain limitations. The first limitation was the use of data from varied sources viz. SRS and NFHS in the same analysis. This was owing to unavailability of maternal health indicators in SRS data and unavailability of mortality rates in NFHS-5 (2019–2021) data. Even if sampling and random errors are likely to occur because of differential sources, the sources were consistent for all units analyzed, states in this case. The analysis was aggregated at the state level and risk of MMR was adjusted for the population, geographical variation and the proportions of maternal health indicators from NFHS-5 (2019–2021) data which enabled the adopted model to produce robust and reliable results. In order to strengthen the utility of statistical models, a single data source with comprehensive indicators although considered ideal is seldom available. The spatial analysis was undertaken at the state level as the availability of data on maternal mortality in India at district level has been the key barrier for quantitative research for policy formulation for preventing maternal deaths in India. With the progress made thus far, we need to identify districts that are lagging. The findings presented in this study are based on the analysis of aggregated data for 19 SRS states. With the availability of district level estimates of MMR in future, the present study may be extended to districts level analysis for identifying the effect of individual districts within states as hot-spots and cold-spots for explaining variation in MMR and associated maternal and health system level indicators. Association of age, socio-economic factors and educational status with MMR is well explored and hence we refrained from considering these factors in our analysis.

To conclude, although India has made significant progress, we need to accelerate our pace to achieve the SDG goal. Better implementation of national programs in Assam, Bihar, and Jharkhand, calls for innovative approaches to improve coverage. States such as Haryana and West Bengal, which shows rising MMR trends and the Bayesian estimation of spatio-temporal effect, need to analyze their programs more deeply. We should gear up to having region specific and district specific strategies to take the interventions to the last mile. Effective monitoring mechanisms and implementation strategies needs to be relooked in the light of these findings.

### Supplementary Information

Supplementary Information.

## Data Availability

Data is available from link: https://censusindia.gov.in/nada/index.php/catalog/42687. Govt of India. Ministry of Health and Family Welfare. Report 2019; 13: 315–322. National Health Accounts Technical Secretariat N. National Health Account Estimates for India, 2018–19. The Healthcare Manager: NABH accreditation statistics, https://expresshealthcaremanagement.blogspot.com/2019/06/nabh-accreditation-statistics.html. https://dhsprogram.com/data/available-datasets.cfm. https://qps.nhsrcindia.org/national-quality-assurance-standards.

## Code availability

Code for the analysis was built in R version 4.3.2. Code will be shared as per personal request.
