# Supplementary material for: Contributing factors for reduction in maternal mortality ratio in India
Source: Sci Rep. 2024 Jun 27;14:14883. doi: 10.1038/s41598-024-65009-0 (PMC11211505; doi:10.1038/s41598-024-65009-0)
Supplement: Supplementary file 1 — Supplementary Information. [file 41598_2024_65009_MOESM1_ESM.docx]

**Supplementary File**

45% decline in MMR is Calculated as [(MMR in 2010 – MMR in 2020)/MMR in 2010] *100.

**Supplementary Table 1: Health system level Indicators used in the analysis.**

| **Sr. No** | **Name of Variable** | **Data description and source** |
| --- | --- | --- |
| **1.** | Health index | The Health Index was be established by NITI Aayog, MoHFW, and the World Bank as an annual systematic tool to encourage States to implement multifaceted measures that will improve health outcomes. We consider estimates from latest health index report[47] (2019) for 19 SRS states. |
| **2.** | Government Health Expenditure | Government health expenditure (GHE) includes spending under all programmes sponsored and managed by the federal, state, and local governments, as well as quasi-governmental organisations and contributors. We have considered estimates of government and out of pocket health expenditure from sixth round of NHA report[48] (2018-19) for 19 SRS states |
| **3.** | Out of Pocket Expenditure | Out of pocket expenditures (OOPE) are costs that families pay out of pocket for medical services. This shows how much financial protection households have against paying for healthcare. Data is from sixth round of NHA report[48] (2018-19) for 19 SRS states. |
| **4.** | Janani Suraksha Yojna beneficiary | Janani Suraksha Yojna (JSY) is the largest conditional cash transfer(CCT) programme in India[25]. The program's overall objective is to lower maternal and newborn mortality and morbidity by improving access to safe pregnancy and delivery services under the National Health Mission. Percentage of women receiving any amount JSY scheme for each of the 19 SRS states is computed from NFHS-5 (2019-2021) data[16];Continuous figures in percentages. |
| **5.** | Number of Accredited Hospitals | A constituent board of the Quality Council of India, the National Accreditation Board for Hospitals & Healthcare Providers (NABH) was established to create and manage an accreditation programme for healthcare organisations. The accreditation criteria for hospitals place a strong emphasis on patient safety and the calibre of services provided by the hospitals. We took state-wise status of number hospitals with their accreditation status as on June 2019[49] |
| **6.** | Number of National Quality Assurance Standards (NQAS) certified hospitals | NQAS were created with the specific needs of public health facilities as well as international best practises in mind for District hospitals, CHCs, PHCs, and Urban PHCs. Standards are primarily designed to help providers evaluate their own performance for room for improvement through established benchmarks and to prepare their facilities for certification. The eight "Areas of Concern" are service provision, patient rights, inputs, support services, clinical care, infection control, quality management, and outcome. We undertook number of NQAS certified hospitals[50] for 19 SRS states; NHSRC website |

**Supplementary Table 2: Maternal Health Indicators used in the analysis.**

| **Sr. No** | **Name of Variable** | **Data description and source** |
| --- | --- | --- |
| **1.** | ANC | The percentage of women aged 15-49 in who received ANC in any state among the sample taken as per NFHS-5 (2019-21) [9] |
| **2.** | PNC | The percentage of women aged 15-49 in who received ANC in any state among the sample taken as per NFHS-5 (2019-21) [9] |
| **3.** | BMI | The percentage of women aged 15-49 for whom BMI was less than 18.5 kg/m^2^ among the sample taken as per NFHS-5 (2019-21) [9] |
| **4.** | Birth Order and Birth Interval | The percentage of women aged 15 - 49 with birth order greater than 3 and the percentage of women aged 15 - 49 with birth interval less than two years in any state among the sample taken as per NFHS-5 (2019 – 21) [9] |
| **5.** | Contraception | Percentage of women who use any contraceptive method among the women, currently married women, and sexually active unmarried women aged 15-49. Sample taken as per NFHS-5 (2019-21) [9] |
| **6.** | C-section | Percentage of Women aged 15-49 delivering through Caesarean section in any state mong the sample taken as per NFHS-5 (2019-21) [9] |

**Supplementary figure 1: Spatial categorisation of SRS and Non-SRS states of India**


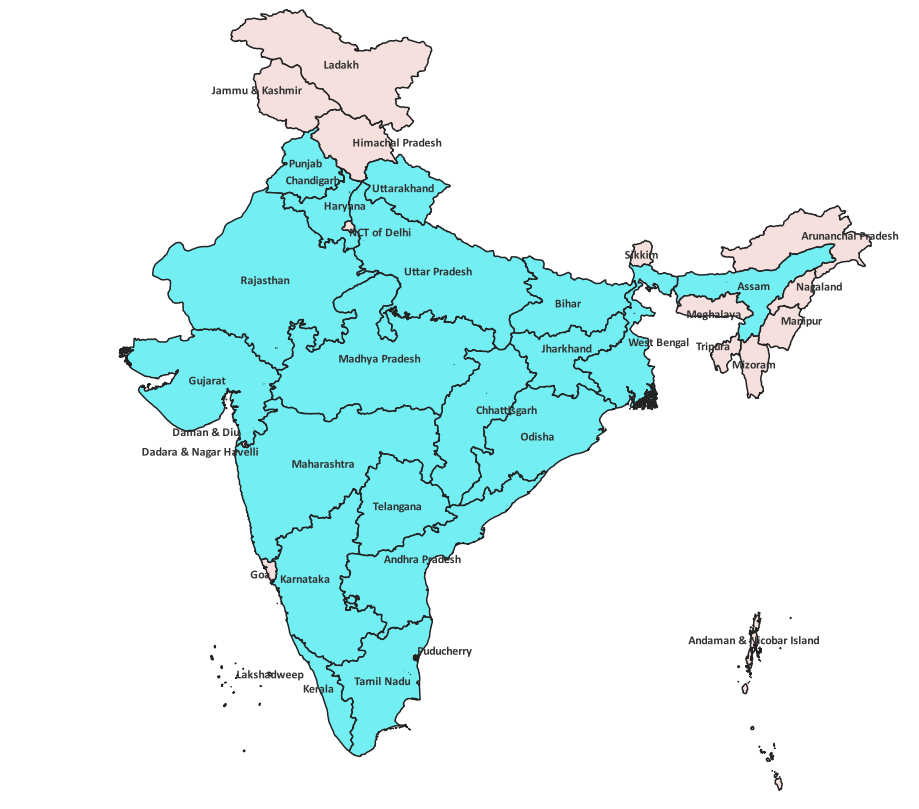


SRS States

Non - SRS States

|  |  |
| --- | --- |
